# An efficient CRISPR/Cas9‐based genome editing system for alkaliphilic *Bacillus* sp. N16‐5 and application in engineering xylose utilization for d‐lactic acid production

**DOI:** 10.1111/1751-7915.14131

**Published:** 2022-08-16

**Authors:** Shiyong Huang, Yanfen Xue, Cheng Zhou, Yanhe Ma

**Affiliations:** ^1^ State Key Laboratory of Microbial Resources, Institute of Microbiology Chinese Academy of Sciences Beijing China; ^2^ University of Chinese Academy of Sciences Beijing China

## Abstract

Alkaliphiles are considered more suitable chassis than traditional neutrophiles due to their excellent resistance to microbial contamination. Alkaliphilic *Bacillus* sp. N16‐5, an industrially interesting strain with great potential for the production of lactic acid and alkaline polysaccharide hydrolases, can only be engineered genetically by the laborious and time‐consuming homologous recombination. In this study, we reported the successful development of a CRISPR/Cas9‐based genome editing system with high efficiency for single‐gene deletion, large gene fragment deletion and exogenous DNA chromosomal insertion. Moreover, based on a catalytically dead variant of Cas9 (dCas9), we also developed a CRISPRi system to efficiently regulate gene expression. Finally, this efficient genome editing system was successfully applied to engineer the xylose metabolic pathway for the efficient bioproduction of d‐lactic acid. Compared with the wild‐type *Bacillus* sp. N16‐5, the final engineered strain with XylR deletion and AraE overexpression achieved 34.3% and 27.7% increases in xylose consumption and d‐lactic acid production respectively. To our knowledge, this is the first report on the development and application of CRISPR/Cas9‐based genome editing system in alkaliphilic *Bacillus*, and this study will significantly facilitate functional genomic studies and genome manipulation in alkaliphilic *Bacillus*, laying a foundation for the development of more robust microbial chassis.

## Graphical abstract

In this study, we reported the successful development of a CRISPR/Cas9‐based genome editing system and a CRISPRi system to efficiently regulate gene expression in alkaliphilic Bacillus sp. N16‐5. Finally, this efficient genome editing system was successfully applied to engineer the xylose metabolic pathway for the efficient bioproduction of D‐lactic acid..

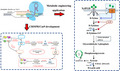

## INTRODUCTION

Alkaliphiles are defined as a group of microorganisms that grow optimally at pH values over 9, usually between pH 10 and 12, and consist of two main physiological groups of microorganisms: alkaliphiles and halo‐alkaliphiles (Horikoshi, 1999). Alkaliphiles require an alkaline pH over 9 for their growth, whereas haloalkaliphiles require both an alkaline pH (over 9) and high salinity (up to 33% [wt/vol] of NaCl). Alkaliphilic *Bacillus* strains are of industrial importance because they can produce various alkaline extracellular enzymes exhibiting great potential in industrial applications (Fujinami & Fujisawa, 2010; Fujiwara et al., 1993; Horikoshi, 1971; Ito et al., 1998; Takami et al., 1989). Additionally, alkaliphilic *Bacillus* strains are promising metabolite producers, such as carotenoids (Aono & Horikoshi, 1991), cholic acid derivatives (Kimura et al., 1994), 2‐phenylamine (Hamasaki et al., 1993) and organic acids (Assavasirijinda et al., 2016; Paavilainen et al., 1994).

Alkaliphile‐based next‐generation industrial biotechnology (NGIB), which allows open nonsterile fermentation owing to the inhibition of traditional microorganisms in high salinity and alkaline environments, is a promising technology that is expected to contribute to building a sustainable bioeconomy (Chen & Jiang, 2017). *Bacillus* sp. N16‐5 is a recalcitrant alkaliphiles strain which exhibits an excellent ability to grow over a broad range of pH (8.5–11.5) and NaCl concentrations (0%–15%) (Li et al., 2011). Additionally, this strain possesses a broad variety of carbon metabolic pathways (Song et al., 2013), and secretes various extracellular polysaccharide hydrolases for degradation of polysaccharides (e.g., xylan, mannan and pectin) (Li et al., 2010; Ma et al., 2004; Zhang et al., 2010). Thus, it has great potential to be developed as an alkaliphilic microbial chassis for production of alkaline enzymes, biopolymers and metabolites with NGIB (Yin et al., 2015).

Optically pure lactic acid (LA), consisting of either L‐LA or D‐LA, is widely used to produce polylactic acid (PLA), which is widely used as biodegradable polyester (Tsuji, 2007; Tsuji et al., 2006). To date, many microorganisms have been used as producers of lactic acid, including fungi, *Lactobacillus* species, *Bacillus* species and various genetically modified strains (Abdel‐Rahman et al., 2021; Mazzoli et al., 2014; Mendes et al., 2021; Mitsui et al., 2020; Poudel et al., 2016; Tsuge et al., 2019). As a potential industrial strain, the alkaliphilic *Bacillus* sp. N16‐5 possesses obvious advantages for lactic acid production, including simple nutrition requirements, high tolerance to sodium salt, non‐sterile fermentation, simple maintenance of stock cultures and utilization of various sugars including lignocellulosic sugars (Assavasirijinda et al., 2016; Li et al., 2010; Song et al., 2013). Lignocellulosic biomass, as the most abundant non‐food resource, can be hydrolysed completely to generate considerable amounts of glucose and xylose. As the second most abundant sugar, the xylose utilization plays an extremely significant role in the efficient bioconversion of lignocellulosic biomass (Wang et al., 2010). Thus, improving the xylose metabolism of alkaliphilic *Bacillus* sp. N16‐5 will be of great advantage for the reduction of the production cost of lactic acid.

Nevertheless, genetic manipulation of alkaliphilic *Bacillus* sp. N16‐5 has been difficult to date, although many conventional *Bacillus* bacteria can be manipulated genetically and easily. Currently, suicide plasmid‐mediated two‐step homologous recombination can only be employed to engineer *Bacillus* sp. N16‐5 (Connelly et al., 2004). However, this traditional method is time‐consuming and has a low efficiency, taking a month to construct a single‐gene mutant. Furthermore, it is barely feasible to perform both large gene fragment deletion and gene pathway insertion. Therefore, to develop the efficient genetic tools for alkaliphilic *Bacillus* sp. N16‐5 are urgently imperative.

Since 2013, the type II CRISPR/Cas9 system from *Streptococcus pyogenes* is the most widely used and well characterized (Choi & Lee, 2016), and has already been employed in many traditional neutrophils, including *Escherichia coli* (Jiang et al., 2015), *Saccharomyces cerevisiae* (DiCarlo et al., 2013), *Bacillus subtilis* (Altenbuchner, 2016), *Streptomyces* (Cobb et al., 2015), *Corynebacterium glutamicum* (Cho et al., 2017) and *Rhodococcus* (Liang et al., 2020). Nevertheless, this system has not been used in the halo‐alkaliphiles of *Bacillus* bacteria except *Halomonas* spp. (Qin et al., 2018) thus far. In addition to the genome editing, the CRISPR/Cas9 system also can be applied to reversibly regulate gene transcription with a catalytically inactive Cas9 (Cas9 with D10A and H840A, dCas9) in many organisms. When performing a transcriptional repression, the dCas9 binds to promoter or open reading frame regions under guiding of sgRNA to prevent RNA polymerase (RNAP) binding or elongation, respectively, known as CRISPR interference (CRISPRi) (Bikard et al., 2013; Qi et al., 2013). To convert dCas9 into a transcription activator, some effectors such as RNAP ω subunit can be used to fuse into dCas9 to stabilize the binding of RNAP to a promoter (Bikard et al., 2013).

In this study, we successfully developed a CRISPR/Cas9‐based genome editing method in alkaliphilic *Bacillus* sp. N16‐5 with an all‐in‐one plasmid system for efficient genome editing, including single‐gene deletion, large gene fragment deletion and exogenous gene insertion. We also harnessed CRISPR/dCas9 to efficiently regulate gene expression. As a proof of concept, we successfully engineered the xylose metabolism of *Bacillus* sp. N16‐5 via this system, generating an efficient chassis strain for the bioproduction of d‐lactic acid from xylose. While this genome editing method is not a great breakthrough for *Bacillus* genomic editing, it really provides a dramatically improved alternative gene editing strategy enabling facile genome modification. Additionally, this study will be of significance for fundamental and applied research in alkaliphilic *Bacillus* sp.

## EXPERIMENTAL PROCEDURES

### Strains, media and culture conditions

The strains and plasmids used in this study are listed in Tables S1 and S2 in the supplemental material. *E. coli* DH5α was used for plasmid construction. Lysogeny broth (10 g/L tryptone, 5 g/L yeast extract, 10 g/L NaCl) was used for the cultivation of *E. coli*; when necessary, 100 μg/ml ampicillin was added. *Bacillus* sp. N16‐5 (CGMCC No. 0369) was isolated from the sediment of Wudunur Soda Lake in Inner Mongolia, China. It was grown aerobically at 37°C and 220 rpm in Horikoshi‐I medium (10 g/L glucose, 5 g/L yeast extract, 5 g/L peptone, 1.31 g/L K_2_HPO_4_•3H_2_O, 0.2 g/L Mg_2_SO_4_•7H_2_O, 20 g/L NaCl). The pH was adjusted to approximately 10.0 by adding sterilized 10% (w/v) Na_2_CO_3_. The medium was also used as a seed culture. Neutral complex medium (NCM) and SA5 medium were used for protoplast transformation of *Bacillus* sp. N16‐5 (Gao et al., 2011). SA5 medium (pH 7.5) consisted of 5% agar, 0.5 M sodium succinate, 10 mM Tris base, 0.5% casamino acid, 0.5% yeast extract, 30 mM MgCl_2_, 12.5 mM CaCl_2_, 1% NaCl and 0.5% glucose. NCM consisted of 5 g/L peptone, 2 g/L yeast extract, 0.34 g/L citric acid, 5 g/L glucose, 0.05 g/L Mg_2_SO_4_•7H_2_O and 20 g/L NaCl and was supplied with 22.8% K_2_HPO_4_•3H_2_O sterilized separately to 1/10 of the total volume. The fermentation medium for studying xylose utilization contained 5 g/L xylose, 2 g/L yeast extract, 3 g/L peptone and 1 g/L Na_2_CO_3_. When needed, 5 μg/mL chloramphenicol (Cm) was added.

### Construction of plasmids

For construction of the all‐in‐one editing plasmid, the *cas9* gene from *S. pyogenes* was first amplified via PCR using the primers cas9‐F and cas9‐R from the pHT01‐*cas9* plasmid. The amplified fragment was inserted into the pMK4‐P_galac_ plasmid to construct the pMK4‐P_galac_‐Cas9 plasmid. Then, the constitutive promoter P_43_ (free of ribosome binding site) was used to express the sgRNA, and the DNA fragment of P_43_‐BbsI/BbsI‐sgRNA was synthesized and then amplified with primers sgRNA‐F and sgRNA‐R, which were ligated into pMK4‐P_galac_‐Cas9 to produce pCas9‐sgRNA. For the quick and easy insertion of the N20 guide sequence into the sgRNA cassette, 20‐nt complementary primers were annealed, and the double‐stranded fragments were cloned into BbsI‐digested pCas9‐sgRNA by T4 DNA ligase.

For homologous recombination repair of DNA double‐strand breaks (DSBs), homologous repair templates flanking target genes were amplified via PCR, and then the purified DNA fragments were assembled into pCas9‐sgRNA via Gibson Assembly according to the product instructions supplied. For construction of the CRISPR/dCas9 editing plasmid, the plasmid pCas9‐sgRNA was used as a template for the mutation of Cas9. To obtain the double mutated dCas9 quickly, one smaller PCR fragment was amplified by primers dCas9‐F1 and dCas9‐R1 with mutations D10A and H840A, respectively, and then the remaining section of the template was amplified with primers dCas9‐F2 and dCas9‐R2. Finally, two DNA fragments were assembled to produce plasmid pdCas9‐sgRNA via Gibson Assembly.

All the DNA primers used in this study are listed in Table S3. DNA polymerases were purchased from TaKaRa (Dalian, China), and restriction enzymes and T4 DNA ligases were purchased from NEB (England). DNA purification kits, gel extraction kits and plasmid extraction kits were purchased from Omega (USA). Gibson Assembly kits for plasmid construction were purchased from Vazyme (Nanjing, China). DNA sequencing was performed by GENEWIZ (Suzhou, China).

### Transformation and mutants screening

Plasmid transformation of *Bacillus* sp. N16‐5 was carried out by protoplast transformation according to previous research (Gao et al., 2011). For preparation of protoplast, the *Bacillus* sp. N16‐5 was cultivated to an OD_600_ of 1.0, and then the collected pellet was washed once with 10 ml ice‐cooled SMMP buffer and suspended in SMMP. Lysozyme was added to the suspension with a final concentration of 0.2 mg/ml and mixed by gently shaking. The suspension was incubated at 37°C for 60 min and then the protoplast collection was washed once with SMMP and suspended in SMMP and stored at −80°C for no more than 1 week. After protoplast transformation of plasmid and regeneration of protoplasts, only one positive colony was picked into the Horikoshi‐I media containing 0.5% glucose and Cm. When the OD_600_ reached 0.8, a final concentration of 1% sugar (xylan, galactose or pectin) or 10% NaCl was added as an inducer to trigger Cas9 expression for DNA cutting. After overnight induction, the culture was plated on the antibiotic‐free plate and cultured overnight at 37°C. Positive mutants were screened by colony PCR. Additionally, the PCR products were sequenced to further confirm successful gene knockout.

### Plasmid curing

To cure plasmids, the plasmid‐harbouring mutant was inoculated into free‐antibiotic Horikoshi‐I medium and subcultured (approximately twice every 12 h). Then, the culture was streaked on an agar plate. Colonies were carefully picked and dotted on both Horikoshi‐I‐C_m_ and Horikoshi‐I agar plates (replica plating). The colonies susceptible to C_m_ were picked and propagated in 1 ml of free‐antibiotic Horikoshi‐I medium. The desirable mutation within the clean mutant (plasmid of which was cured) was further confirmed through PCR. The plasmid curing efficiency was calculated by dividing the number of C_m_‐sensitive colonies by the number of tested colonies.

### Growth measurement

The wild‐type strain *Bacillus* sp. N16‐5 and mutants were inoculated from frozen stock at −80°C into seed medium and precultured at 37°C and 200 rpm for 12 h. After that, 5 ml of the resulting culture were inoculated into 50 ml new seed medium with subsequent cultivation overnight. Samples were taken periodically to monitor the growth conditions every 2 h. All experiments were performed in triplicate.

### Quantitative real‐time PCR (qRT‐PCR)

For total RNA extraction, *Bacillus* sp. N16‐5 was cultured in Horikoshi‐I overnight. After 4–6 h of cultivation, total RNA was extracted using the HiPure Bacteria RNA Kit (Magen, Guangzhou, China). Reverse transcription was performed using HiScript III qRT SuperMix (with gDNase) (Vazyme, Nanjing, China). qPCR analysis was conducted using the SuperReal Premix SYBR green kit (Tiangen Biotech, Beijing, China) based on 16S rRNA as the reference and the housekeeping gene *ispH* as a positive control. Relative quantities were calculated using the 2^−ΔΔCt^ method (Schmittgen & Livak, 2008). All experiments were performed in triplicate.

### Fluorescence measurement

Overnight cultures of *Bacillus* sp. N16‐5 were transferred to fresh Horikoshi‐I media. After cultivation at 37°C and shaking at 200 rpm for 24 h, the cells were harvested by centrifugation at 5000 × g for 10 min, washed once and resuspended in PBS buffer (pH 7.4). The red fluorescent protein (RFP) fluorescence value was detected using a microplate reader (SpectraMax M5, Molecular Devices) with the wild‐type strain *Bacillus* sp. N16‐5 as a negative control. The excitation and emission wavelengths were 550 and 574 nm respectively. The fluorescence intensities were normalized to the OD_600_. All experiments were performed in triplicate.

### Fermentation and analysis of metabolites

The nonsterile green fermentation process was used for the production of d‐lactic acid according to a previous method (Assavasirijinda et al., 2016). For lactic acid production, *Bacillus* sp. N16‐5 and mutant were inoculated in 100‐mL flasks containing 50 ml of xylose media and incubated at 37°C under static conditions. Samples were taken periodically, and the concentrations of d‐lactic acid, residual glucose and xylose were determined.

The biomass concentration was calculated from OD_600_ values according to a reported method (Meiswinkel et al., 2013), in which one OD_600_ unit is equal to 0.25 g/L cell dry weight (CDW). Glucose, xylose and organic acids in the culture supernatants were quantified using an Agilent 1260 HPLC system equipped with an RID detector and an Aminex HPX‐87H cation exchange column (BioRad, USA). The samples were first filtered through a 0.22‐μm filter, loaded onto a column operated at 60°C and eluted with 18 mM H_2_SO_4_ at a flow rate of 0.5 ml/min. All experiments were performed in triplicate.

## RESULTS AND DISCUSSION

### Construction of the CRISPR/Cas9 system in *Bacillus* sp. N16‐5

**FIGURE 1:**
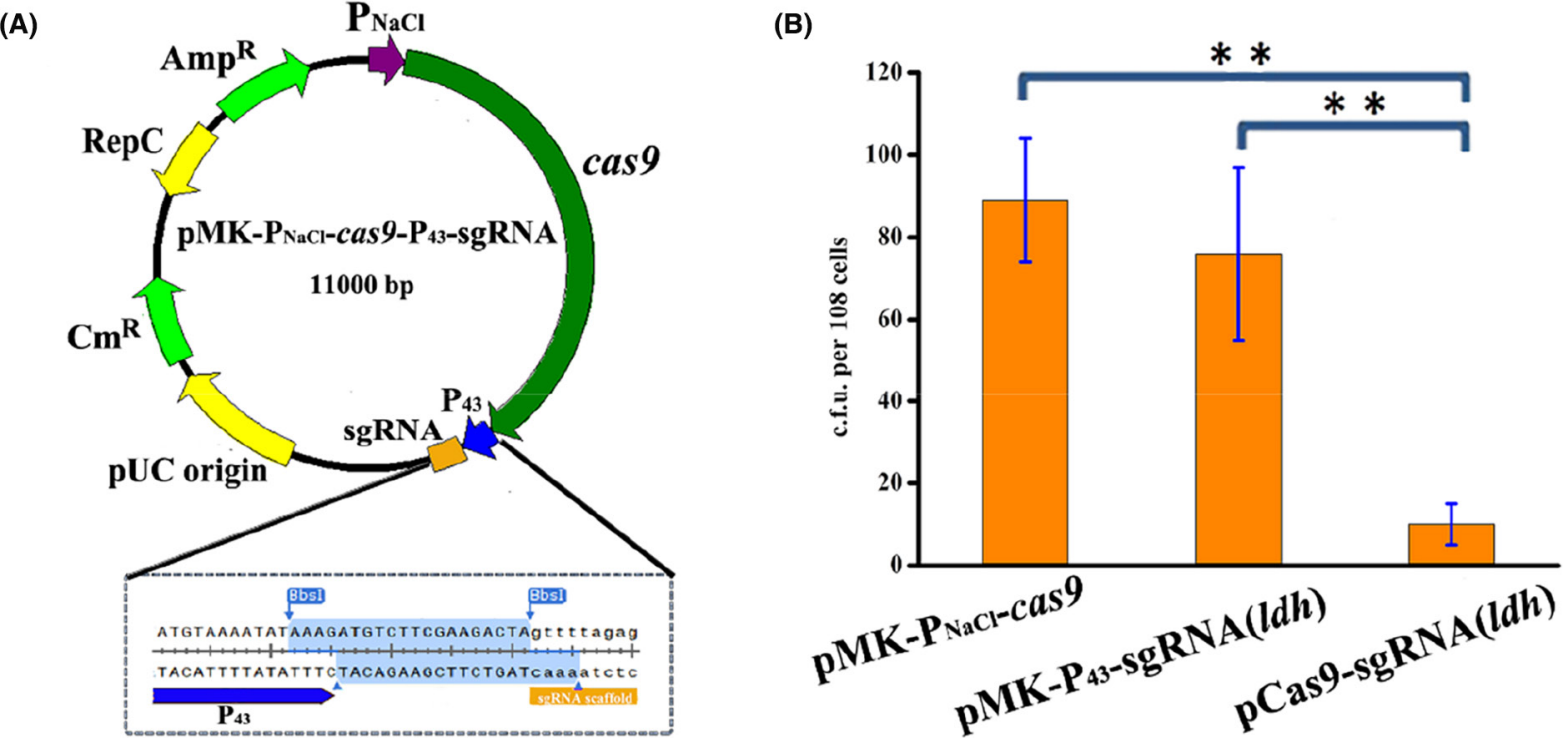
Construction of functional Cas9‐sgRNA complex in *Bacillus* sp. N16‐5. (A) Plasmid map of pMK‐P_NaCl_‐Cas9‐P_43_‐sgRNA. (B) Determination on cleavage activity of Cas9‐sgRNA complex, in which pMK‐P_NaCl_‐Cas9‐P_43_‐sgRNA(*ldh*) contained sgRNA targeting ldh with pMK‐P_NaCl_‐Cas9 and pMK‐P_43_‐sgRNA(*ldh*) as control. Error bars indicate the standard deviation from three independent biological replicates, the ** indicates *p* < 0.01 relative to the control.

The type II CRISPR/Cas9 system from *Streptococcus pyogenes* is widely used for genome editing due to the inherent simplicity and flexibility in sequence requirements for sgRNA. Although many similar CRISPR/Cas9 systems have been development for *Bacillus* species (Hartz et al., 2021; Wu et al., 2020), whether they can work well still faced some uncertainty when employed directly in our strain *Bacillus* sp. N16‐5 due to the applicability promoters and compatibility and stability of plasmids. Therefore, we need to develop a new CRISPR/Cas system in *Bacillus* sp. N16‐5. First, we designed and constructed an all‐in‐one plasmid system that integrated Cas9 with the gRNA into the pMK4 plasmid available for *Bacillus* sp. N16‐5 (Gao et al., 2011) (Figure 1A). Considering the toxicity of Cas9 to some bacteria, such as *C. glutamicum* (Cho et al., 2017) and *Halomonas* (Qin et al., 2018), we chose a NaCl‐sensitive promoter (from *Bacillus* sp. N16‐5) with an adjustable expression level (Figure S1) for the expression of Cas9 and the strong constitutive promoter P_43_ for the expression of sgRNA. The sgRNA‐encoding sequence P_43_‐BbsI/BbsI‐sgRNA was synthetized and then ligated into the plasmid for rapid and easy exchange of the spacer sequence by the addition of two BbsI sites, in which the spacer sequence was generated by annealing single‐stranded oligonucleotide primers and then ligated into the BbsI site (Figure 1A). After constructing the editing plasmid pCas9‐sgRNA, we will investigate whether the CRISPR/Cas9 system can induce double‐strand breaks (DSBs) at the targeted genomic site of *Bacillus* sp. N16‐5. To this aim, the *ldh* gene responsible for L‐lactate production was chosen as the test case, and then the plasmid pCas9‐sgRNA(*ldh*) (sgRNA sequence described in Table 1) as well as the control plasmids pMK‐P_NaCl_‐Cas9 and pMK‐P_43_‐sgRNA(*ldh*) were constructed. All three plasmids above were introduced individually into *Bacillus* sp. N16‐5 by protoplast transformation according to a reported method (Gao et al., 2011) and the positive colonies were cultivated in liquid medium with 100 g/L NaCl for inducible expression of Cas9. Colony numbers decreased by 88% when pCas9‐sgRNA(*ldh*) was introduced into the *Bacillus* sp. N16‐5 compared to that of the control plasmid either pMK‐P_NaCl_‐Cas9 or pMK‐P_43_‐sgRNA(*ldh*) (Figure 1B), which indicated that the CRISPR/Cas9 system developed in this study successfully induced DSBs at the *ldh* locus with high functionality. However, the transformation of pCas9‐sgRNA(*ldh*) into *Bacillus* sp. N16‐5 can still generate few transformants (12 ± 3 per 10^8^ cells) escaping the cleavage of the Cas9/sgRNA complex, which might result from either DSBs repair by non‐homologous end joining (NHEJ) that exist in some *Bacillus* species (Weller et al., 2002) or mutation of the Cas9/sgRNA complex (Liang et al., 2020). That was further investigated in our following research.

**TABLE 1:** Genome editing with CRISPR/Cas9 system in *Bacillus* sp*.* N16‐5

	Target site	gRNA Guide Sequence^a^	Deletion length (bp)	Editing plasmid^b^	Mutant/Total colony	Editing efficiency
Gene deletion	*ldh*	GTTGGAGCTGTTATGGACAG	991	pSY07	10/10	100%
	ORF‐2686	TTTGGATAACCCTACGGTTG	1714	pSY09	(5/8)	62.5%
	ORF‐2642	GCTGATATACCGTACTGAAG	1954	pSY10	(7/10)	70%
	ORF‐4112	TCTGTCACTGCATTTACTGG	2460	pSY11	(10/10)	100%
	ORF‐2635	GTCGGCTGAAGGTATTGCTG	2324	pSY12	(10/10)	100%
	ORF‐3943	GTAATCCGCTCCTTTAGTTG	1309	pSY13	(3/4)	75%
	ORF‐2646	GGGCTGAGCAATGTCCTTAT	1466	pSY14	(7/10)	70%
	ORF‐212	GTCCTTGATAAACTGCCATA	1200	pSY15	(9/10)	90%
	ORF‐581	GTCCTTGATAAACTGCCATA	2205	pSY16	(7/12)	58%
	ORF‐3563	GTAATAAATGAGACGATCAA	1506	pSY17	(10/10)	100%
	ORF‐2639	GAAGATGCAGCAGAATCTCT	1113	pSY18	(10/10)	100%
Large DNA fragment deletion	flagellum	GCAGTGGGACAACACTTCCA	5000	pSY20	(9/9)	100%
		GCAGTGGGACAACACTTCCA	10,000	pSY22	(8/10)	80%
		GCAGTGGGACAACACTTCCA	26,000	pSY24	(5/10)	50%
*rfp* insertion	ORF‐3943	GTAATCCGCTCCTTTAGTTG	1309	pSY27	(8/9)	88.9%
*cas9* insertion	*phage*	GCTCGCCCCAATATCAATGG	315	pSY28	(5/10)	50%
(*alsS‐alsD*) insertion	L*‐ldh*	GTTGGAGCTGTTATGGACAG	500	pSY29	(5/8)	62.5%

^a^ The guide sequence was designed using a web‐based tool developed by previous report (Park et al., 2015).

^b^ The detailed information about editing plasmids can be founded in Table S1 of Supplementary materials.

In bacteria, DSBs can be repaired by NHEJ or homology‐directed repair (HDR). NHEJ pathways, usually present in very few bacteria, such as *Streptomyces coelicolor* (Tong et al., 2015) and *Mycobacterium smegmatis* (Sun et al., 2018), can repair DSBs when no templates for homologous recombination are present. Although the genes encoding protein Ku and LigD responsible for NHEJ could also be found in *Bacillus* sp. N16‐5, RT‐qPCR showed that neither *ku* nor *ligD* was transcribed (Figure S2). An identical result was also found in *Clostridium cellulolyticum* (Xu et al., 2015). In addition, we further analysed the colonies escaping Cas9 cleavage by Sanger DNA sequencing. None of the six sequenced colonies showed any mutations near the target site of sgRNA in the genome (results not shown), indicating that the survival of these colonies did not result from NHEJ but from possible mutations in either the P_galac_‐Cas9 or P_43_‐sgRNA expression cassette sequence as well as the off‐target of sgRNA. Taken together, there is no functional NHEJ system in the *Bacillus* sp. N16‐5 and CRISPR/Cas9 system developed in this study was highly functional, which laid the foundation for subsequent efficient genome editing by HDR.

### Optimization of the CRISPR/Cas9 system and single‐gene deletion in *Bacillus* sp. N16‐5

**FIGURE 2:**
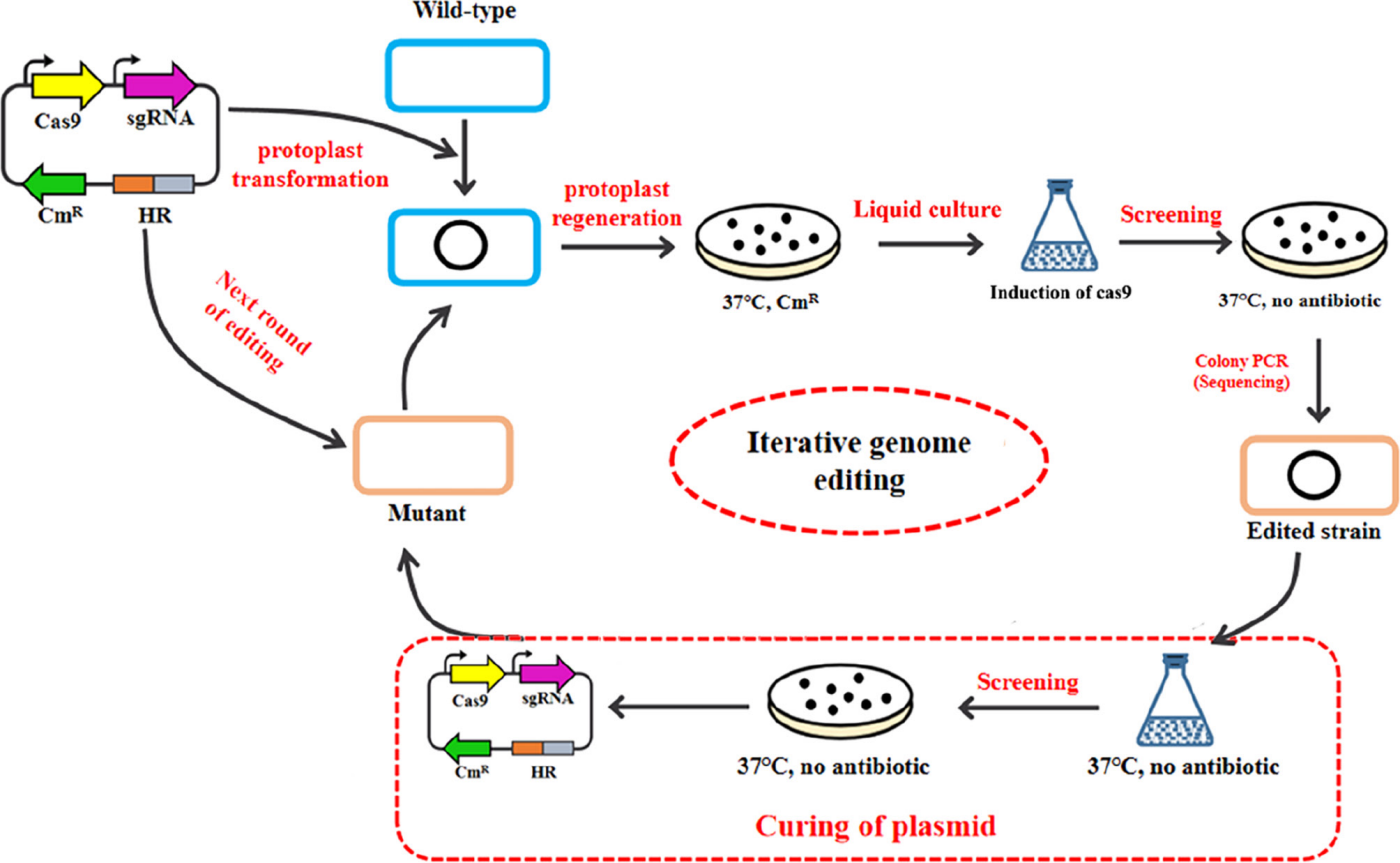
Procedure of CRISPR/Cas9 genome editing in *Bacillus* sp. N16‐5. The editing plasmid for a targeted gene was transformed into cells and then some of positive colonies were picked onto a plate to induce genome editing. Positive mutants are identified by colony PCR and verified by sequencing. For the iterative genome editing, the mutant will be cultivated in antibiotic‐free media to cure the editing plasmid, and the subsequent strain would be used for the next round of editing.

**FIGURE 3:**
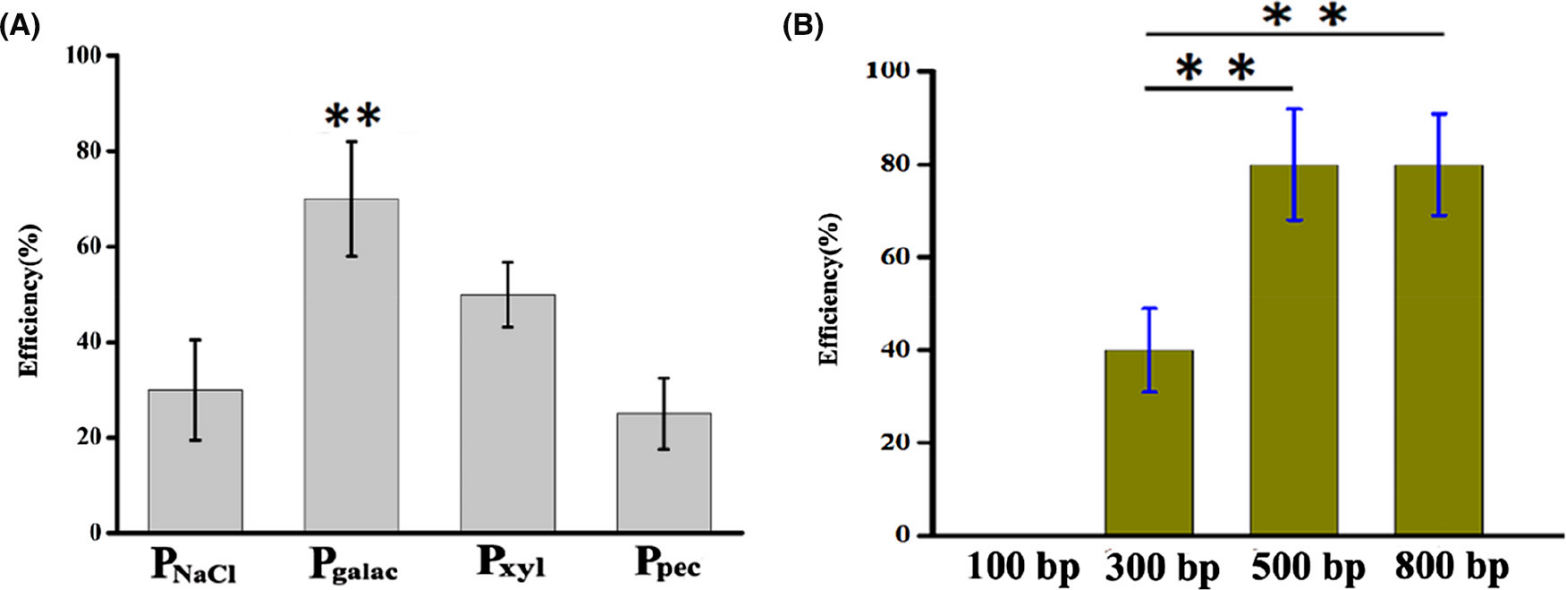
The optimization of CRISPR/Cas9 system. (A) Effect on editing efficiency by expression of *cas9* under different promoters. (B) Effect of length‐different homologous recombination templates on deletion efficiency. Editing efficiency is calculated as number of mutants/Total colonies selected. Error bars indicate the standard deviation from three independent biological replicates, the ** indicates *p* < 0.01 relative to the control.

As mentioned above, the CRISPR/Cas9 system was established successfully via validation of Cas9 cleavage. Subsequently, the native L‐lactate dehydrogenase gene *ldh* was selected to examine the genome editing of CRISPR/Cas9 coupled with HDR in *Bacillus* sp. N16‐5, the procedure of genome editing is shown in Figure 2. Meanwhile, to explore the effect of the expression intensity of Cas9 on the editing efficiency, different sugar‐inducible promoters were also used to express Cas9 (Figure S3). The deletion efficiency of *ldh* was 29%, 65%, 47% and 22% with the promoters P_NaCl_, P_galac_, P_xyl_ and P_pec_ respectively (Figure 3A). Compared with other inducible promoters, the galactose‐inducible promoter P_galac_ was able to generate higher editing efficiency when expressing Cas9, which suggested that the appropriate expression of Cas9 protein will contribute to high gene editing efficiency (Qin et al., 2018). Considering the possible toxicity of Cas9, we also analysed its effect on the cell growth of *Bacillus* sp. N16‐5. The expression of Cas9 with different inducible promoters showed no effect on strain growth (Figure S4). Accordingly, the expression of Cas9 seemed to be harmless to *Bacillus* sp. N16‐5, which was consistent with the report about the strain *Rhodococcus ruber* TH (Liang et al., 2020). Thus, the promoter P_galac_ will be used for the subsequent construction of editing plasmids.

Furthermore, different homology arm lengths (on each side of the target site) were also studied to evaluate the effect on editing efficiency. Although previous research indicated that longer homology arms will offer higher editing efficiency for the CRISPR/Cas9 system (Jiang et al., 2017), it will still be challenging to construct an all‐in‐one CRISPR/Cas9 editing plasmid (usually reaching 14 kb) with 1 kb homology arms and transform it into a strain with high efficiency (Jiang et al., 2017). To determine the optional homology arm length, *ldh* was chosen as a target site. When the homology arm length was 100 bp, 300 bp, 500 bp and 800 bp, the corresponding gene deletion efficiencies were 0%, 40%, 80% and 80% respectively (Figure 3B). Obviously, a high deletion efficiency of 80% can be obtained even with a 500 bp homology arm, which indicated that *Bacillus* sp. N16‐5 naturally harbours the efficient RecA‐dependent homologous recombination system. Different from other prokaryotes usually need an additional recombination system for an enhanced editing efficiency due to the deficiency of native RecA‐dependent homologous recombination system, such as *E. coli* that require the λ‐Red recombination system (Jiang et al., 2015) or *C. glutamicum* that use RecT recombinase (Cho et al., 2017) and *R. ruber* TH that use Che9c60&61 recombinase (Liang et al., 2020), *Bacillus* sp. N16‐5 can complete high editing efficiency only rely on native homologous recombination system to efficiently repair the DSBs caused by Cas9. Thus, 500 bp of the homologous arm was chosen for subsequent genome editing.

Taken together, an efficient CRISPR/Cas9 system for genome editing in *Bacillus* sp. N16‐5 was established based on a pMK4 plasmid carrying the native galactose‐inducible promoter P_galac_ for the expression of Cas9 and a strong constitutive promoter P_43_ for the expression of sgRNA coupled with a 500 bp homologous arm for recombination repair. However, when the editing plasmid was transformed into *Bacillus* sp. N16‐5 by protoplast transformation, only a small number of colonies can be obtained on the plate after single transformation (maximum 20–50 cfu/μg DNA), which was significantly difficult to obtain the edited colonies from such inadequate candidate colonies (< 50 cfu every single transformation). To solve this question above, this study provided a new method that one positive colony after protoplast transformation was cultivated in liquid culture, and Cas9 was not induced to initiate the editing process until it proliferated into a large population, which has already been proved in genome editing of *E. coli* (Feng et al., 2018). The procedure was as follows (Figure 2): first, the expression cassettes of *cas9* and sgRNA were cloned into plasmid pMK4 to construct an all‐in‐one parental plasmid pCas9‐sgRNA, and then pCas9‐sgRNA can be used to construct any editing plasmid by cloning corresponding 20 nt spacers and homology arms. Next, the editing plasmid was transformed via protoplast transformation into *Bacillus* sp. N16‐5 strain, which was screened via protoplast regeneration plates with chloramphenicol resistance to obtain positive colonies. Only one positive colony was picked into Horikoshi‐I media until OD_600_ reached 0.8. At this time, galactose with a final concentration of 1% was added to the medium to induce Cas9 expression overnight to produce DSBs. Two pairs of primers near the genomic editing site were designed to verify mutants using colony PCR, and the target site of the mutants was sequenced for further confirmation. Finally, the mutant was cultivated in antibiotic‐free media to cure the editing plasmid for iterative genome editing. This two‐step genome editing strategy bypassed the issues that the low protoplast transformation efficiency of *Bacillus* sp. N16‐5 resulted in low genome editing efficiency.

After that, the CRISPR/Cas9‐based genome editing system was employed to perform single‐gene deletion. As summarized in Table 1, different chromosomal sites were targeted for successful deletion, with editing efficiency ranging from 62.5% to 100%. These results indicate that single‐gene deletion by the CRISPR/Cas9 system developed in this study is site‐independent and has high deletion efficiency. Therefore, the CRISPR/Cas9 system can accurately perform single‐gene deletion with high efficiency in *Bacillus* sp. N16‐5.

### Large DNA fragment deletion using the CRISPR/Cas9 system

**FIGURE 4:**
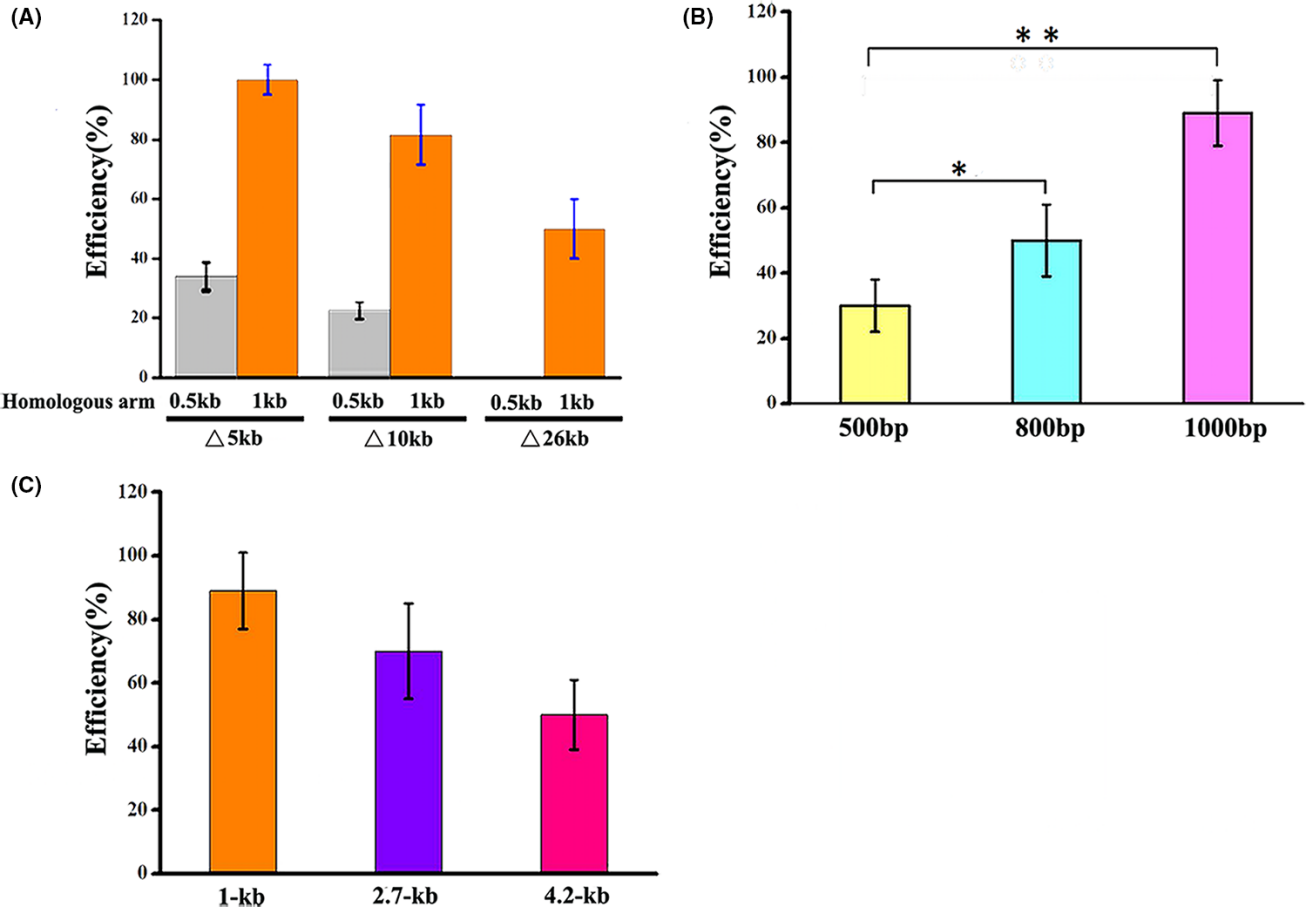
Multiplex genome editing in *Bacillus* sp. N16‐5 using the CRISPR/Cas9 system. (A) Editing efficiency of different size deletion with homologous arm length. (B) Editing efficiency of *rfp* (714 bp) insertion with different homologous arm length. (C) Editing efficiency of different‐size exogenous genes insertion. Error bars indicate the standard deviation from three independent biological replicates, the * and ** indicate *p* < 0.05 and 0.01 relative to the control respectively.

Previous studies have shown that large genomic deletions in *Bacillus* sp. play an important role in heterologous enzyme expression, genome reduction (Westers et al., 2003), strain improvement (Thwaite et al., 2002) and overproduction of antibiotics (Zobel et al., 2015). To date, although large gene cluster deletion by CRISPR/Cas9 has already been performed in several traditional *Bacillus* bacteria, there is no report on large fragment deletion of alkalophilic *Bacillus* sp. In this research, we successfully achieved the deletion of single genes with size within 3 kb (Table 1) via the CRISPR/Cas9 system with high efficiency. Next, we attempted to delete the larger gene fragments to further explore the robustness of the CRISPR/Cas9 system. As an example, the nonessential 26 kb flagellum gene cluster was chosen as a test case, and then three different‐length DNA fragments were designed to validate the robustness of CRISPR/Cas9‐mediated larger gene fragment deletion. When using a 0.5 kb homologous arm for recombination repair and a single sgRNA for guiding, the efficiency was only 37% and 20% for the deletion of 5 kb and 10 kb DNA fragments respectively. It failed to perform the 26 kb fragment deletion with a 0.5 kb homologous arm (Figure 4A). However, when the homologous arm was extended to 1 kb and equipped with a single sgRNA for guiding of Cas9, the efficiency of the 5 kb, 10 kb and 26 kb fragment deletions was 100%, 80% and 50% respectively (Figure 4A and Table 1). Therefore, it could be concluded that CRISPR/Cas9‐mediated large gene fragment deletion, especially over 5 kb, can still reach high efficiency when using a 1 kb homologous arm as a repair template.

Although some research had completed larger DNA fragment deletion, such as deletion of *bac*ABC (42.7 kb) with an efficiency of 79.0% via CRISPR/Cas9n (the mutant of *Streptococcus pyogenes* Cas9 with D10A that causes single‐strand DNA breaks) in *B. licheniformis* (Li et al., 2018), the deletion of the 38 kb plipastatin‐synthesizing *pps* operon in *B. subtilis* by CRISPR/Cas9 with 80% efficiency (So et al., 2017), all of them mentioned above were accomplished by dual‐plasmid with dual‐sgRNA for guiding cleavage of the genome, which differed from that in our study via the all‐in‐one CRISPR/Cas9 system with single‐sgRNA for guiding cleavage. Expressing multiple gRNAs in a single construct generally requires using the same promoter several times, ultimately causing various issues in terms of much more labour‐intensive cloning, plasmid stability as well as reaching robust transcriptional expression of the gRNAs (Ferreira et al., 2017; Zhang et al., 2019). Therefore, CRISPR/Cas9 in this study has more advantages in large fragment deletion compared with that using dual‐sgRNA for guiding.

### Targeted insertion of exogenous DNA into the genome via CRISPR/Cas9 system

In addition to genomic deletion, insertions of exogenous DNA fragments into the genome are still important for genetic engineering of *Bacillus* sp. N16‐5, especially for metabolic engineering and genomic function research. In this study, to demonstrate the insertion of exogenous genes via CRISPR/Cas9, the red fluorescent protein gene *rfp*, as the reporter gene, under promoter P_43_ for expression, was inserted into the phage‐related locus (ORF‐1754 involving in toxin secretion or phage lysis) to construct the strain SY‐RFP (Figure S5). Additionally, the effect of homologous arm lengths on the insertion efficiency was also investigated. The results showed that the insertion efficiency was 88% when using the 1 kb homologous arm as the repair template, but the efficiency decreased to 50% and 30% when the homologous arm was 800 and 500 bp respectively (Figure 4B), which indicated that the insertion efficiency was reduced following the decrease in homologous arm length. The same results were also found in genome editing of *H. bluephagenesis* (Qin et al., 2018). Therefore, the length of homologous arms was consequently extended to 1 kb to effectively perform gene insertion.

As an application example, the 1 kb D‐lactate dehydrogenase gene D*‐ldhA* from *Lactobacillus delbrueckii* and the 2.7 kb acetoin synthesis genes (*alsS*, *alsD*) from *B. subtilis* 168 were successfully inserted into native L‐*ldhA* locus with insertion efficiencies of 85% and 70% respectively. The 4.2 kb *cas9* gene was successfully inserted into locus encoded phage protein gene ORF‐1754 of *Bacillus* sp. N16‐5 with insertion efficiencies of 50% (Figure 4C). It showed that the insertion efficiency decreased with increasing length of the insertion, and the possible reason was that increasing plasmid capacity caused by incorporation of exogenous genes leads to the low plasmid stability in *Bacillus* sp. N16‐5 and then reduces the insertion efficiency of exogenous genes. To solve this issue, one way is to develop electroporation for plasmid transformation, which allows a linear donor DNA fragment to be transformed into *Bacillus* sp. N16‐5 coupled with an all‐in‐one plasmid. Another is to develop the two‐plasmid CRISPR/Cas9 system, which divides the *cas9* gene and sgRNA expression components into two different plasmids to reduce the plasmid size. The 4‐hydroxybutyrate formation genes with size of 4.5 kb were inserted into the genome of *H. bluephagenesis* TD01 with 50% efficiency by a two‐plasmid CRISPR/Cas9 system (Qin et al., 2018).

Although insertion of larger exogenous DNA fragments into *Bacillus* sp. N16‐5 by the all‐in‐one plasmid CRISPR/Cas9 system was still challenging, we still achieved the maximum 4.2 kb exogenous gene insertion with 50% efficiency in this study, which obviously was better than the previous related researches on gene insertion in *Bacillus* sp (Hao et al., 2020; Wu et al., 2020). Taken together, it appears that insertions of exogenous DNA fragments into *Bacillus* sp. N16‐5 are of great feasibility by CRISPR/Cas9.

### Development of a CRISPR/dCas9 system for gene expression regulation

The catalytically inactive Cas9 mutant (dCas9) has already been proven to be an efficient transcriptional engineering tool for gene interference or activation (Qi et al., 2013; Tong et al., 2015). For transcriptional activation, the native RNAP ω subunit has already been used as an efficient tool for genes activation when fused into dCas9 in *E. coli*, in which dCas9 is directed to the promoter region and recruits the polymerase by the interaction between ω and β’ subunit of RNAP (Bikard et al., 2013). In our study, to construct a dCas9‐mediated multidirectional transcriptional regulation system for both transcriptional activation and repression, the editing plasmid pCas9‐sgRNA was used as a backbone to obtain the plasmid pdCas9‐sgRNA by mutating Cas9 firstly, and then the native RNA polymerase ω subunit was ligated into the pdCas9‐sgRNA to generate the fusion protein dCas9‐ω according to reported research.

**FIGURE 5:**
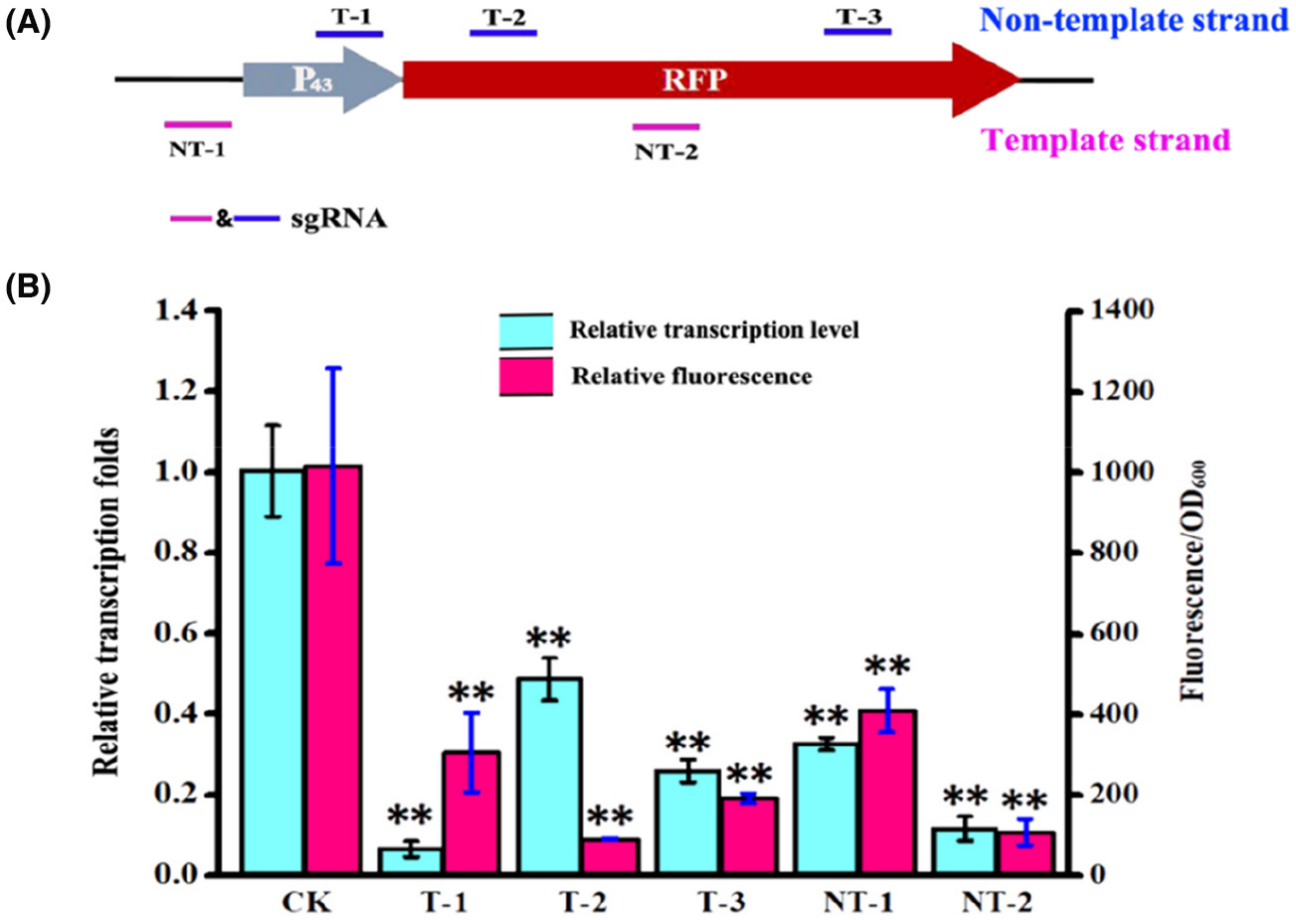
CRISPR/dCas9‐assisted gene transcriptional regulation in *Bacillus* sp. N16‐5. (A) Schematic diagram of sgRNA targeting location. (B) Transcriptional and fluorescence analyses of different targeting location, the relative expression folds were calculated using the 2^−ΔΔCt^ method. All data were the average of three biological replicates with standard deviations, the ** indicates *p* < 0.01 relative to the control.

To evaluate dCas9‐mediated transcriptional regulation, the red fluorescent protein gene *rfp* was used as the reporter, and five gRNAs with different targeting sites located in the P_43_ *‐rfp* expression cassette in strain SY‐RFP were designed to evaluate the effect of targeting sites on transcriptional regulation (Figure 5A). Among them, transcriptional activation was not achieved, and the transcriptional level of *rfp* decreased by 67% when sgRNA targeted 313 bp upstream of the transcription start site of P_43_, which was different from that in *B. subtilis* and possibly resulted from the poor activation ability of the RNA polymerase ω subunit in the *Bacillus* sp. N16‐5. Therefore, to enable the transcriptional activation function of our CRISPR/dCas9 system, more activator proteins need to be screened and evaluated, as in previous reports (Wu et al., 2020). Additionally, the transcriptional repression efficiency can be completed from 51.4% to 89.4% when the sgRNAs specifically target either the non‐template strand or the template strand of the P_43_ *‐rfp* expression cassette, and the highest repression efficiency reached 93.5% when sgRNAs target the promoter region (Figure 5B), which is different from the results in *S. coelicolor* that transcriptional expression can only occur when sgRNA targets the non‐template strand (Tong et al., 2015). We speculated that perhaps the fusion protein dCas9‐ω enhanced the steric blockade of transcription, so the transcriptional repression can occur when sgRNA targets both strands of DNA. Overall, these results indicate that the dCas9‐ω‐based system can efficiently mediate the repression of transcription elongation either by sgRNAs specifically targeting the T strand or the NT strand of the target gene. We believe that the CRISPR/dCas9 system can also be endowed with many functions, such as the study of essential genes and complex regulatory networks, as well as for quick screening of functional genes in *Bacillus* sp. N16‐5.

### Metabolic engineering of *Bacillus* sp. N16‐5 for the production of d‐lactic acid from xylose

As a proof‐of‐concept example in metabolic engineering, we applied the established CRISPR/Cas9 system to engineer the xylose metabolism pathway of *Bacillus* sp. N16‐5 for the efficient production of d‐lactic acid. To obtain the d‐lactic acid‐producing strain, the d‐lactic acid dehydrogenase gene D‐*ldhA* from *L. delbrueckii* was integrated into the native L‐lactic acid dehydrogenase gene (L‐*ldhA*) locus of wild‐type (WT) *Bacillus* sp. N16‐5 to generate strain SY‐ldh which could only produce d‐lactic acid. However, it showed a poor ability on xylose consumption and d‐lactic acid production with xylose as the sole carbon source. Thus, to further enhance xylose consumption, the native xylose metabolism pathway needs to be reengineered.

**FIGURE 6:**
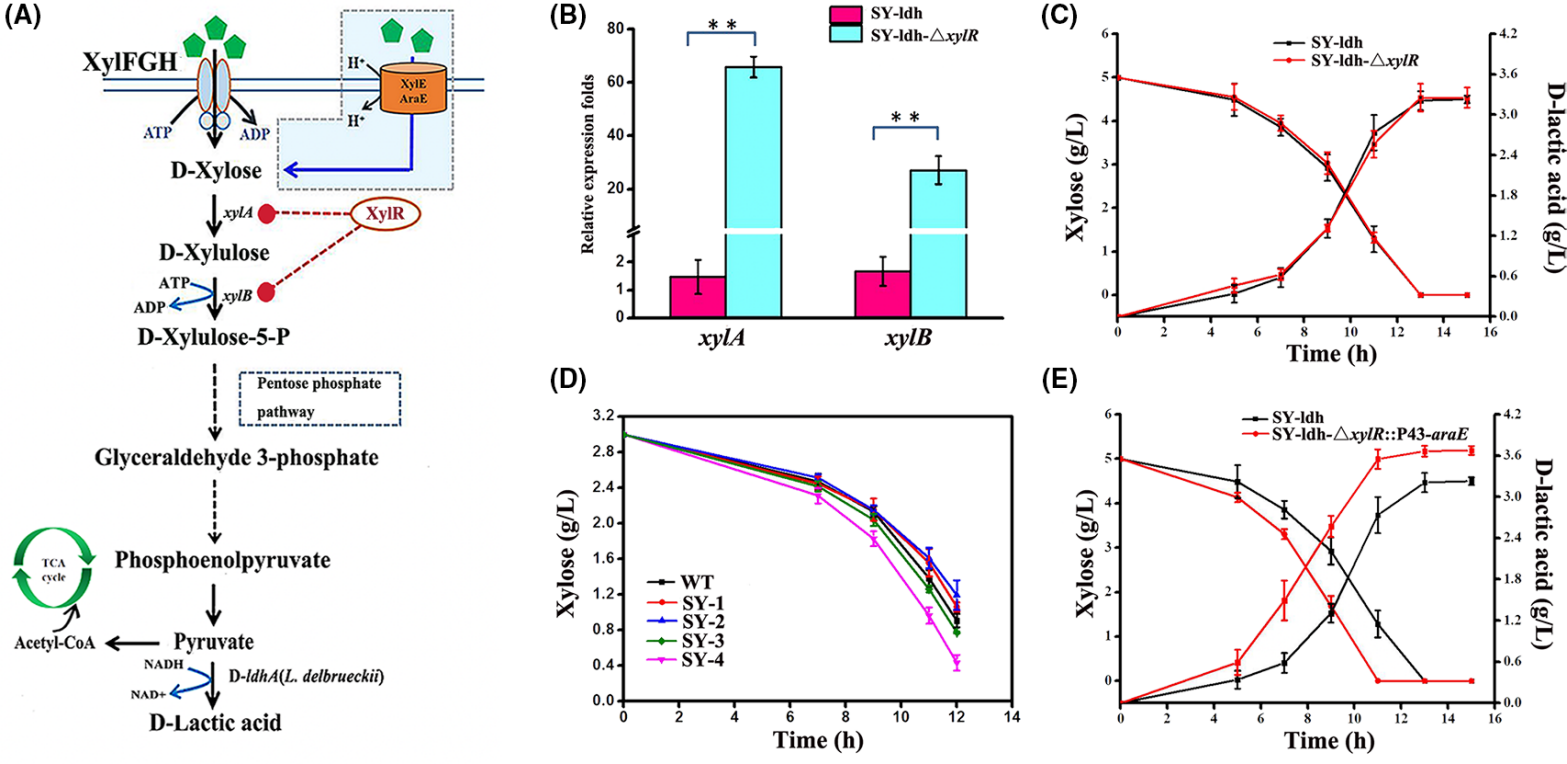
Applying CRISPR/Cas9‐based genome editing system to engineer *Bacillus* sp. N16‐5 for the bio‐production of d‐lactic acid from xylose. (A) Engineering xylose catabolic pathway in *Bacillus* sp. N16‐5. (B) Quantitative RT‐PCR analysis of the transcriptional changes of some key genes (*xylA* and *xylB*) involved in xylose metabolism in strain SY‐ldh‐△*xylR*. (C) The profile of xylose consumption and d‐lactic acid production of strain SY‐ldh and SY‐ldh‐△*xylR*, (D) Time‐course of the xylose fermentation with expressing various xylose transporters in wild‐type strain N16‐5. (E) The profile of xylose consumption and d‐lactic acid production of strain SY‐ldh and SY‐ldh‐△*xylR*::P_43_‐*araE*. All data were the average of three biological replicates with standard deviations, the ** indicates *p* < 0.01 relative to the control.

It has been reported that the xylose metabolism negative regulator XylR could specifically repress the gene expression of the xylose pathway in some Gram‐positive organisms (Figure 6A) (Li et al., 2017; Wen et al., 2020). To enhance xylose utilization in strain SY‐ldh, *xylR* was knocked out via CRISPR/Cas9. The RT‐qPCR results showed that genes involved in the xylose degradation pathway were both significantly upregulated in engineered SY‐ldh‐△*xylR*, in which the relative transcription levels of *xylA* and *xylB* were upregulated by 65‐fold and 27‐fold respectively (Figure 6B). Nevertheless, it was found that the average xylose consumption rate of SY‐ldh‐△*xylR* was 0.338 ± 0.027 g/L/h, which was almost the same as that of SY‐ldh (0.335 ± 0.011 g/L/h) at 11 h (Figure 6C), which was also reported in *B. licheniformis* (Li et al., 2017) and *Clostridium cellulovorans* (Wen et al., 2020). Therefore, we speculate that the low consumption rate of xylose was probably due to low efficiency of xylose transporters in SY‐ldh‐△*xylR*.

Substrate transport is another bottleneck to xylose metabolism in some host strains (Dvořák, & Lorenzo, 2018; Elmore et al., 2020; Gao et al., 2020; Wen et al., 2020). Accordingly, to further enhance xylose utilization in *Bacillus* sp. N16‐5, four xylose transporters, including *xylE* and *araE* from *E. coli*, as well as native *xylF* and *araE* from *Bacillus* sp. N16‐5, were evaluated for their ability to facilitate xylose uptake, resulting in engineered strains SY‐1, SY‐2, SY‐3 and SY‐4 respectively. The time‐course curves of the fermentation showed that the engineered strain SY‐4 expressing native arabinose/proton symporter AraE harboured the highest xylose consumption rate, with a 23.0% increase compared with WT during 12 h fermentation (Figure 6D). The XylE and AraE from *E. coli* failed to increase xylose uptake in this study, although they already have proven the ability to increase xylose uptake in *E. coli* (Bai et al., 2016) and *Pseudomonas putida* (Dvořák, & Lorenzo, 2018; Elmore et al., 2020). This is probably because XylE and AraE need a H^+^ gradient for xylose transport, while a Na^+^ gradient appears to maintain the survival of *Bacillus* sp. N16‐5, which resulted in XylE and AraE not working effectively. After that, for stable expression, *araE* under the P_43_ promoter was integrated into the *xylR* locus of WT to generate SY‐ldh‐△*xylR*::P_43_‐*araE*. The fermentation results showed that the xylose consumption rate and d‐lactic acid productivity of SY‐ldh‐△*xylR*::P_43_‐*araE* were 0.454 ± 0 g/L/h and 0.318 ± 0.013 g/L/h, respectively, which were 34.3% and 27.7% higher than those of the WT respectively (Figure 6E). These results further confirmed our hypothesis that substrate transport can play a significant role in xylose metabolism in *Bacillus* sp. N16‐5. Although the final engineered strain SY‐ldh‐△*xylR*::P_43_‐*araE* reached a high xylose consumption rate and d‐lactic acid productivity, it was still lower than that of some strains (Assavasirijinda et al., 2016; Elmore et al., 2020). Thus, considering the complicated regulatory mechanism of sugar metabolism in *Bacillus* sp. N16‐5 (Song et al., 2013), it will be a good choice to employ the xylose non‐phosphorylative metabolism pathway to enhance the xylose consumption rate to bypass the native sugar metabolism regulation (Bai et al., 2016; Cabulong et al., 2021; Choi et al., 2016; Tai et al., 2016). Currently, PLA production with a complete biological process by one‐step fermentation from renewable resources has been also developed by metabolically engineered bacteria (Huang et al., 2021). Depending on the CRISPR/Cas9 for an efficient genome modification and metabolic engineering, *Bacillus* sp. N16‐5 will also be promising platform for biosynthesis of PLA by the expression of propionyl‐CoA transferase and PHA synthase. Taken together, the enhanced xylose metabolism and d‐lactic acid production have further proven the usefulness of CRISPR/Cas9 in the metabolic engineering of *Bacillus* sp. N16‐5 and further increased the potential of the *Bacillus* sp. N16‐5 as a chassis cell for PLA production.

## CONCLUSIONS

In summary, the CRISPR/Cas9‐based genome editing system and CRISPR/dCas9‐based transcriptional regulation system were both developed for efficient genome editing in alkaliphilic *Bacillus* sp. N16‐5, including single‐gene deletion, large gene fragment deletion, gene insertion and transcriptional repression. This CRISPR/Cas9‐based genome editing system was developed based on an all‐in‐one plasmid, which is beneficial for rapid plasmid curing and subsequent iterative genome editing. In addition, in the face of the low efficiency of gene editing caused by the low efficiency of protoplast transformation, the two‐step method for the genome editing process was employed to successfully avoid this problem, which also provided a reference for the development of the CRISPR/Cas9 system in non‐model microorganisms with low transformation efficiency. As an application in metabolic engineering, this CRISPR/Cas9 system was used to reengineer the xylose metabolism pathway in *Bacillus* sp. N16‐5 for d‐lactic acid production, and the final engineered strain showed 34.3% and 27.7% increases in xylose consumption and d‐lactic acid production respectively. This study will speed up research on *Bacillus* sp. N16‐5 as a halo‐alkaliphile cell factory for the efficient production of high value products.

## AUTHOR CONTRIBUTIONS

S.H. contributed to the conceptualization, data curation, formal analysis, investigation, methodology, software, validation, visualization, writing—original draft and writing—review and editing. C.Z. and Y.X. contributed to the conceptualization, funding acquisition, project administration, visualization, writing—review and supervision. Y.M. contributed to the funding acquisition, project administration and supervision.

## FUNDING INFORMATION

This work was financially supported by the National Key R&D Program of China (No. 2020YFA0906800) and the National Natural Science Foundation of China (No. 31870789).

## CONFLICT OF INTEREST

The authors declare that they have no competing interests.

## Supporting information


**Appendix S1** Supporting information
