# Supplementary material for: An efficient CRISPR/Cas9‐based genome editing system for alkaliphilic Bacillus sp. N16‐5 and application in engineering xylose utilization for d‐lactic acid production
Source: Microb Biotechnol. 2022 Aug 16;15(11):2730–43. doi: 10.1111/1751-7915.14131 (PMC9618316; doi:10.1111/1751-7915.14131)
Supplement: Supplementary file 1 — Appendix S1 Supporting information [file MBT2-15-2730-s001.docx]

**An efficient CRISPR/Cas9-based genome editing system for** **alkaliphilic *Bacillus* sp. N16-5 and application in engineering xylose utilization for D-lactic acid production**

Shiyong Huang^1,2^, Yanfen Xue^1^, Cheng Zhou^1*^, Yanhe Ma^1*^

^1^ State Key Laboratory of Microbial Resources, Institute of Microbiology, Chinese Academy of Sciences, Beijing 100101, China;

^2^ University of Chinese Academy of Sciences, Beijing 100049, People’s Republic of China.

* Co-corresponding authors:

Cheng Zhou, Tel: +86 10 64807618; Fax: +86 10 64807616; E-mail: [zhoucheng@im.ac.cn](mailto:zhoucheng@im.ac.cn)

Yanhe Ma, Tel: +86 10 64807590; Fax: +86 10 64807616; E-mail: mayanhe@im.ac.cn

**Table S1 Plasmids used in this study.**

| Plasmid | Relevant characteristics | Source |
| --- | --- | --- |
| pMK4 | *E.coli- Bacillus* shuttle vector; AmpR, CmR, ColE1 origin in *E. coli;* CmR, pC194 origin in *Bacillus* sp. | (A.Sullivan *et al.*, 1984) |
| pHT01-cas9 | *E.coli- Bacillus* shuttle vector; *cas9* gene | (Lu *et al.*, 2019) |
| pCas9-sgRNA | pMK4 derivate, editing plasmid with cas9 and sgRNA scaffold. | This study |
| pCas9-sgRNA(*ldh*) | pMK4 derivate, sgRNA targeting *ldh* | This study |
| pMK-P_NaCl_-Cas9 | pMK4 derivate, *cas9* under promoter P_NaCl_ | This study |
| pMK-P_43_-sgRNA(*ldh*) | pMK4 derivate, *cas9* under promoter P_NaCl_, sgRNA targeting *ldh* under promoter P_43_ | This study |
| pSY01 | pMK4 derivate, *cas9* under promoter P_NaCl_, sgRNA targeting *ldh* under promoter P_43_, △*ldh* (991bp) 1000 bp donor | This study |
| pSY02 | pMK4 derivate, *cas9* under promoter P_xyl_, sgRNA targeting *ldh* under promoter P_43_, △*ldh* (991bp) 1000 bp donor | This study |
| pSY03 | pMK4 derivate, *cas9* under promoter P_galac_, sgRNA targeting *ldh* under promoter P_43_, △*ldh* (991bp) 1000 bp donor | This study |
| pSY04 | pMK4 derivate, *cas9* under promoter P_pec_, sgRNA targeting *ldh* under promoter P_43_, △*ldh* (991bp) 1000 bp donor | This study |
| pSY05 | pMK4 derivate, *cas9* under promoter P_galac_, sgRNA targeting *ldh* under promoter P_43_, △*ldh* (991bp) 100 bp donor | This study |
| pSY06 | pMK4 derivate, *cas9* under promoter P_galac_, sgRNA targeting *ldh* under promoter P_43_, △*ldh* (991bp) 300 bp donor | This study |
| pSY07 | pMK4 derivate, *cas9* under promoter P_galac_, sgRNA targeting *ldh* under promoter P_43_, △*ldh* (991bp) 500 bp donor | This study |
| pSY08 | pMK4 derivate, *cas9* under promoter P_galac_, sgRNA targeting *ldh* under promoter P_43_, △*ldh* (991bp) 800 bp donor | This study |
| pSY09 | pMK4 derivate, *cas9* under promoter P_galac_, sgRNA targeting ORF-2686, 500 bp donor | This study |
| pSY10 | pMK4 derivate, *cas9* under promoter P_galac_, sgRNA targeting ORF-2642, 500 bp donor | This study |
| pSY11 | pMK4 derivate, *cas9* under promoter P_galac_, sgRNA targeting ORF-4112, 500 bp donor | This study |
| pSY12 | pMK4 derivate, *cas9* under promoter P_galac_, sgRNA targeting ORF-2635, 500 bp donor | This study |
| pSY13 | pMK4 derivate, *cas9* under promoter P_galac_, sgRNA targeting ORF-3943, 500 bp donor | This study |
| pSY14 | pMK4 derivate, *cas9* under promoter P_galac_, sgRNA targeting ORF-2646, 500 bp donor | This study |
| pSY15 | pMK4 derivate, *cas9* under promoter P_galac_, sgRNA targeting ORF-212, 500 bp donor | This study |
| pSY16 | pMK4 derivate, *cas9* under promoter P_galac_, sgRNA targeting ORF-581, 500 bp donor | This study |
| pSY17 | pMK4 derivate, *cas9* under promoter P_galac_, sgRNA targeting ORF-3563, 500 bp donor | This study |
| pSY18 | pMK4 derivate, *cas9* under promoter P_galac_, sgRNA targeting ORF-2639, 500 bp donor | This study |
| pSY19 | pMK4 derivate, *cas9* under promoter P_galac_, sgRNA targeting flagellum, △*ldh* (5kb) 500 bp donor | This study |
| pSY20 | pMK4 derivate, *cas9* under promoter P_galac_, sgRNA targeting flagellum, △*ldh* (5kb) 1000 bp donor | This study |
| pSY21 | pMK4 derivate, *cas9* under promoter P_galac_, sgRNA targeting flagellum, △*ldh* (10kb) 500 bp donor | This study |
| pSY22 | pMK4 derivate, *cas9* under promoter P_galac_, sgRNA targeting flagellum, △*ldh* (10kb) 1000 bp donor | This study |
| pSY23 | pMK4 derivate, *cas9* under promoter P_galac_, sgRNA targeting flagellum, △*ldh* (26kb) 500 bp donor | This study |
| pSY24 | pMK4 derivate, *cas9* under promoter P_galac_, sgRNA targeting flagellum, △*ldh* (26kb) 1000 bp donor | This study |
| pSY25 | pMK4 derivate, *cas9* under promoter P_galac_, sgRNA ORF-3943, △ORF-3943 (1309bp) :: P_43_-*rfp*, 500 bp donor | This study |
| pSY26 | pMK4 derivate, *cas9* under promoter P_galac_, sgRNA ORF-3943, △ORF-3943 (1309bp) :: P_43_-*rfp*, 800 bp donor | This study |
| pSY27 | pMK4 derivate, *cas9* under promoter P_galac_, sgRNA ORF-3943, △ORF-3943 (1309bp) :: P_43_-*rfp*, 1000 bp donor | This study |
| pSY28 | pMK4 derivate, *cas9* under promoter P_galac_, sgRNA ORF-3943, △*phage*(315bp) :: P_galac_-*cas9*, 1000 bp donor | This study |
| pSY29 | pMK4 derivate, *cas9* under promoter P_galac_, sgRNA ORF-3943, △*ldh* (500bp) :: P_xyl_-*alsS-alsD*, 1000 bp donor | This study |
| pSY30 | pMK4 derivate, dCas9 (Cas9 with D10A and H840A) under promoter P_galac_, sgRNA under promoter P_43_, used for gene expression regulation | This study |
| pSY31 | pSY30 derivate, dCas9 under promoter Pgalac, sgRNA under promoter P_43_, targeting the template strand of P_43_-*rfp*, T-1 | This study |
| pSY32 | pSY30 derivate, dCas9 under promoter Pgalac, sgRNA under promoter P_43_, targeting the template strand of P_43_-*rfp*, T-2 | This study |
| pSY33 | pSY30 derivate, dCas9 under promoter Pgalac, sgRNA under promoter P_43_, targeting the non-template strand of P_43_-*rfp*, NT-1 | This study |
| pSY34 | pSY30 derivate, dCas9 under promoter Pgalac, sgRNA under promoter P_43_, targeting the upsream of P_43_-*rfp*, UP-1 | This study |
| pSY35 | pSY30 derivate, dCas9 under promoter Pgalac, sgRNA under promoter P_43_, targeting the upsream of P_43_-*rfp*, UP-2 | This study |
| pSY36 | pMK4 derivate, *cas9* under promoter P_galac_, sgRNA(*xylR*), △*xylR* (583bp), 1000 bp donor | This study |
| pSY37 | pMK4 derivate, *cas9* under promoter P_galac_, sgRNA(*xylR*), △*xylR* (583bp) :: P_43_-*araE*, 1000 bp donor | This study |
| pSY38 | pMK4 derivate, *cas9* under promoter P_galac_, sgRNA(*ldh*), △*ldh*(288bp) :: D-*ldhA*, 1000 bp donor | This study |

**Table S2 Important strains used in this study.**

| Strain | Relevant characteristics | Source |
| --- | --- | --- |
| *E. coli* DH5α | Plasmid construction, F -φ80 lacZΔM15Δ（lacZYA-argF）U169 end A1 recA1 hsdR17(rk -,mk -) sup E44 λ- thi-1 gyrA96 relA1 phoA | Tsingke Biotechnology Co., Ltd, China. |
| Engineered *Bacillus* sp*.* N16-5 | | |
| N16-5 | A facultative alkaliphilic strain, producing multiple extracellular hydrolases and organic acids | China General Microbiological Culture Collection Center |
| SY-ldh | N16-5 with *ldhA* gene replaced by a D-*ldhA* gene | This study |
| SY-ldh-△xylR | SY-ldh with *xylR* gene knocked out | This study |
| SY-RFP | N16-5 with phage-related gene ORF-1754 replaced by P_43_-*rfp* cassette | This study |
| SY-1 | N16-5 with plasmid pMK-P_ldh_-Ec*xylE* | This study |
| SY-2 | N16-5 with plasmid pMK-P_ldh_-Ec*araE* | This study |
| SY-3 | N16-5 with plasmid pMK-P_ldh_-*xylF* | This study |
| SY-4 | N16-5 with plasmid pMK-P_ldh_-*araE* | This study |
| SY-5 | SY-ldh with *xylR* gene replaced by P_43_-*araE* expression cassette | This study |

**Table S3 Main primers used in this study.**

| **Name** | **Sequence** | **Description** | |
| --- | --- | --- | --- |
| cas9F | TTAGAAAGGAGGTAATTTAGatggacaagaagtacagcatc | Application of cas9 (4784 bp) | |
| cas9R | ctaAtccgggatccCCCGGGTTAgtcgcctcccagctgag |  |  |
| sgRNA-F | AATTCCGTGACAAGGGATCCTATTTTTTTGCCAAAGCTGTAATG | Application of P43-BbsI/BbsI-sgRNA (426 bp) | |
| sgRNA-R | tgggaggcgacTAACCCGGGggatcccggaTtagtccaag |  |  |
| dCas9-F1 | ACGATAGCGTCCACATCGTAGTC | Application of a short fragment of cas9 with mutations D10A and H840A (2500 bp) | |
| dCas9-R1 | TGGCCATCGGCACCAACTCTGTGGGC |  |  |
| dCas9-F2 | AGAGTTGGTGCCGATGGCCAGGCCGATGCTGTAC | Application of vector fragment (8592 bp) | |
| dCas9-R2 | TACGATGTGGACGCTATCGTGCCTCAGAGC |  |  |
| **Single-gene deletion (just partial display)** | | | |
| Ldh-up-F | CACACAGGAAACAGCTATGACGTCGTAATTGATGGCACGG | Deletion of L*ldh* | |
| Ldh-up-R | CAGCCCTTTAGAAATTCATTATCTGTCTTGCCTATTGTACAC |  |  |
| Ldh-down-F | ATAATGAATTTCTAAAGGGCTG |  |  |
| Ldh-down-R | CTGCAGCCAAGCTTGGCGTATGCGATACTCACTCCTAGTAAG |  |  |
| 2642-Up-F | CACACAGGAAACAGCTATGATACCTGGACCAACTGTCGCG | Deletion of ORF-2642 |  |
| 2642-Up-R | CAGCTGGTAGCTGGCTATTTGGAGTATCAATGAACGGCATGAG |  |  |
| 2642-down-F | CCAAATAGCCAGCTACCAGCTG |  |  |
| 2642-down-R | CTGCAGCCAAGCTTGGCGTAATGGCTAGCTTGTCTAGCCG |  |  |
| 4112-up-F | AGGCACCCCAGGCTTTACACCCATGTAACTAATAGCGTACG | Deletion of ORF-4112 |  |
| 4112-up-R | CTGTATTGAACTTGAGGTCAGAGCATTAGACAGCTAGTTG |  |  |
| 4112-down-F | TGACCTCAAGTTCAATACAG |  |  |
| 4112- down-R | CCAAGCTTGGCGTAATCATGCGCATACGTGCATAAGCTAAG |  |  |
| 3563-up-F | ttgaacttggactagtccggTCAGCAGAAAAAGTAAAAGGTCA | Deletion of ORF-3563 |  |
| 3563-up-R | TCGAATAGGAGGGGGGAGATTTTCTTTGATTCCTGCTATC |  |  |
| 3563-down-F | ATCTCCCCCCTCCTATTCGAG |  |  |
| 3563-down-R | taacaggaattcgggatcTTTAAACATCCTAGTGCAAATTACC |  |  |
| 2639-up-F | ttgaacttggactagtccggGTTGATCGACTTCAGCTTCC | Deletion of ORF-2639 |  |
| 2639-up-R | GGCTGAGCCAGTATTTATTGCAAATGATTGAAGCAAGGGT |  |  |
| 2639-down-F | CAATAAATACTGGCTCAGCC |  |  |
| 2639-down-R | ataacaggaattcgggatcGTACATTCCTTTATTCCAGG |  |  |
| **Large DNA fragment deletion** | | |  |
| (Flagellum-5K)-up-F | AGGCACCCCAGGCTTTACGATCTGATGCGACGACAAAGG | Deletion of 5 kb fragment of flagellum gene cluster |  |
| (Flagellum-5K)-up-R | ATCAATATGCCCAGCAAGCACCCTGTTAAAATAAGCCCGG |  |  |
| (Flagellum-5K)-down-F | TGCTTGCTGGGCATATTGAT |  |  |
| (Flagellum-5K)-down-R | TAGGATCCCTTGTCACGGAAATTCCTCCGCAATAGTTTCT |  |  |
| (Flagellum-10K)-up-F | AGGCACCCCAGGCTTTACACTTGGGAAATTCTAGATGTGG | Deletion of 10 kb fragment of flagellum gene cluster |  |
| (Flagellum-10K)-up-R | ATGTCTATGCCAATCGTCGGCCAAAATGAACGTTGTCCTGTG |  |  |
| (Flagellum-10K)-down-F | CCGACGATTGGCATAGACAT |  |  |
| (Flagellum-10K)-down-R | CCAAGCTTGGCGTAATCATGAGCCTTCGCAATTAGTGAGCTG |  |  |
| (Flagellum-20K)-up-F | AGGCACCCCAGGCTTTACACCCAGAAGGAACATTTCCTC | Deletion of 20 kb fragment of flagellum gene cluster |  |
| (Flagellum-20K)-up-R | CGACAAGAGGTCGATTAGTTTGAGCAGCTCGTGATTTCGC |  |  |
| (Flagellum-20K)-down-F | AACTAATCGACCTCTTGTCG |  |  |
| (Flagellum-20K)-down-R | CCAAGCTTGGCGTAATCATGAGGTAATGAGGGTCTGCTAG |  |  |
| **Insertion of exogenous DNA** | | |  |
| 3943-up-F | AGCTCACTCATTAGGCACCGCTAGCCTCTTTGTTCGTCCATATCG | Insertion of *rfp* in ORF-3943 locus |  |
| 3943-up-R | ATAGAGCGTATCGATCGTTGTAAATC |  |  |
| (P43-RFP)-F | CAACGATCGATACGCTCTATAGCTTCGTGCATGCAGGCCG |  |  |
| (P43-RFP)-R | GAATGCATGAGAGCCGACTTTTATCTATGCCCTAATTTACTAG |  |  |
| 3943-down-F | AAGTCGGCTCTCATGCATTC |  |  |
| 3943-down-R | CTTGTCACGGAATTTCGTACTCAGGCTGTGCTCATGTTTCACGG |  |  |
| phage-up-F | AGGCACCCCAGGCTTTACGATTACGGCACAAGCGCCAGCTAT | Insertion of *cas9* in phage locus |  |
| phage-up-R | ACGGAACAATTTAGACGGGG |  |  |
| (Pgala-Cas)-F | CCCCGTCTAAATTGTTCCGTCGAAAATGTAAAACCGTCTC |  |  |
| (Pgala-Cas)-R | TTAgtcgcctcccagctgagac |  |  |
| phage-down-F | ctcagctgggaggcgacTAAGTGTCAGAAAATCAAGGACG |  |  |
| phage-down-R | ATAGGATCCCTTGTCACGGAACTATGCAAAGACTGTGTTCTTTC |  |  |
| Ldh-up-F | GCTCACTCATTAGGCACCCCAGGCATTAGGTACAGTTATTGC | Insertion of (*alsD-alsS*) in L-*ldh* locus |  |
| Ldh-up-R | CATATCTCTTTAGCTTGACCCTGTCTTGCCTATTGTACAC |  |  |
| Pxyl-alsSD-F | GTGTACAATAGGCAAGACAGGGTCAAGCTAAAGAGATATGTG |  |  |
| Pxyl-alsSD-R | TCATTAACCACCACTCTCCTttattcagggcttccttcag |  |  |
| Ldh-down-F | AGGAGAGTGGTGGTTAATGAG |  |  |
| Ldh-down-R | CAGCCAAGCTTGGCGTAATCGGTTATTAAAGAAGATGGCC |  |  |

Underlined letters represent homologous sequences for fusion PCR or Seamless Cloning.


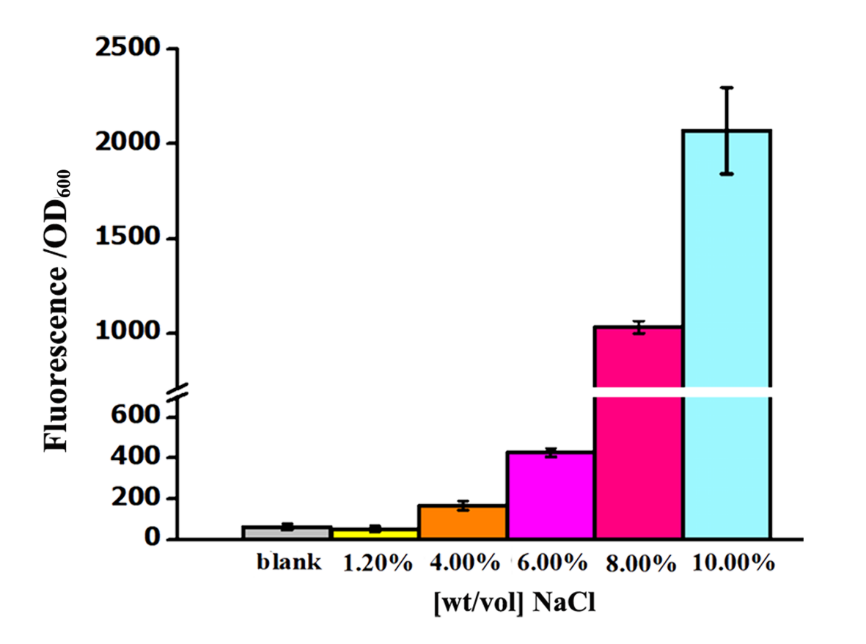


**Fig. S1.** **The RFP fluorescence under NaCl-sensitive promoter with different NaCl concentration.** Experiments were performed in triplicate.


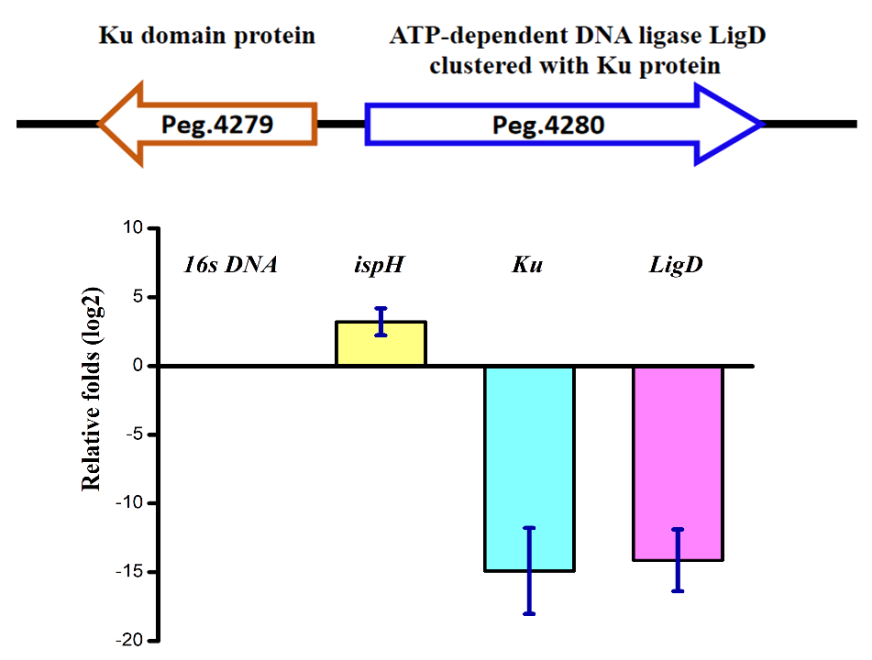


**Fig. S2.** **The transcriptional analysis of predicted NHEJ system in *Bacillus* sp*.* N16-5.** The 16s rDNA used as internal calibrator and house-keeping gene *ispH* as positive control for RT-qPCR analysis and the relative expression folds were calculated using the 2^-ΔΔCt^ method**.**


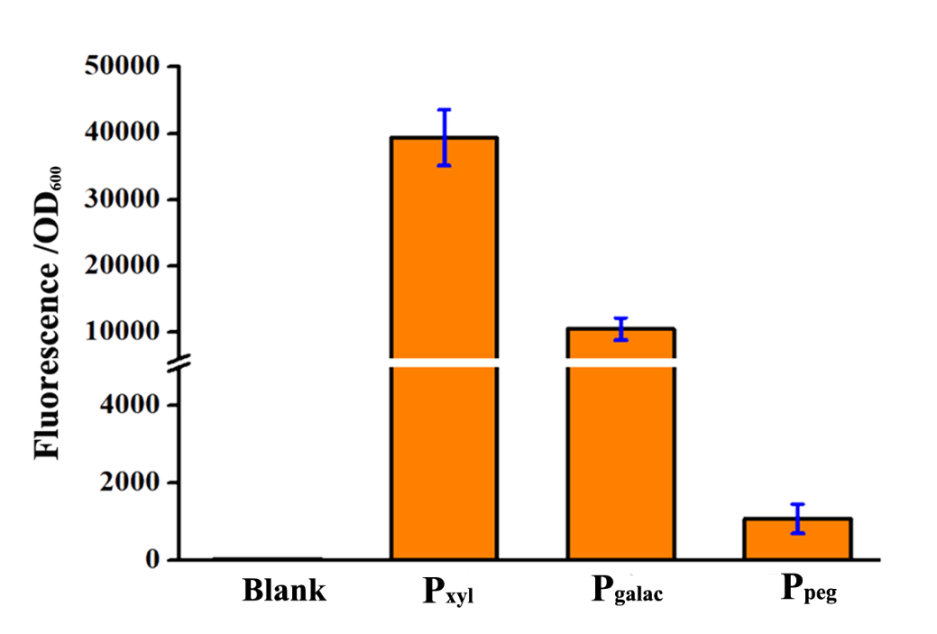


**Fig. S3. The RFP fluorescence under sugar-sensitive promoter with 1% sugar concentration.** Xyl, xylan; galac, galactose; pec, pectin. Experiments were performed in triplicate.


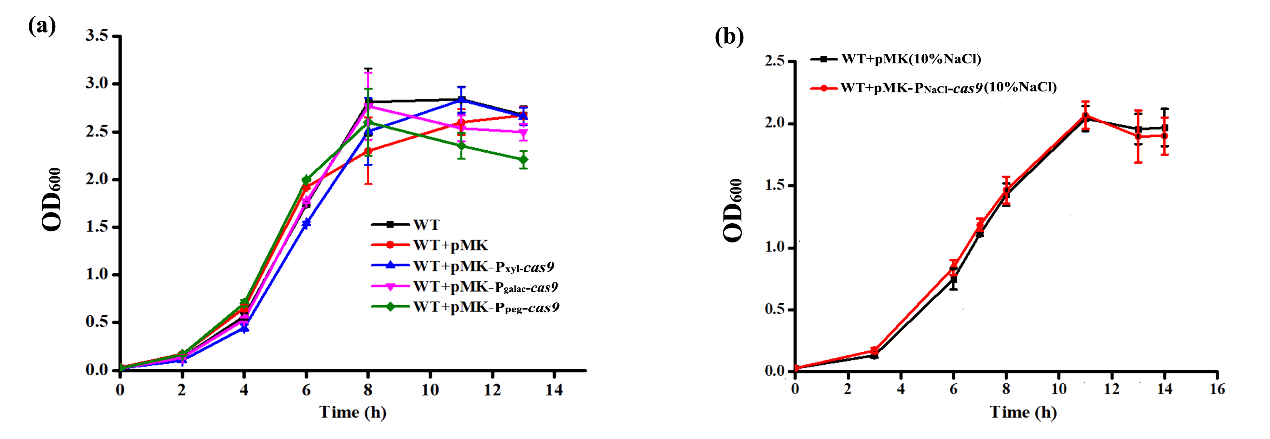


**Fig. S4.** **Effect of Cas9 expression with different promoters on cell growth. (a)** Cas9 was expressed with sugar-sensitive promoters with 1% sugar concentration. Xyl, xylan; galac, galactose; pec, pectin. **(b)** Cas9 was expressed with NaCl-sensitive promoter with 10% concentration. All experiments were performed in triplicate.


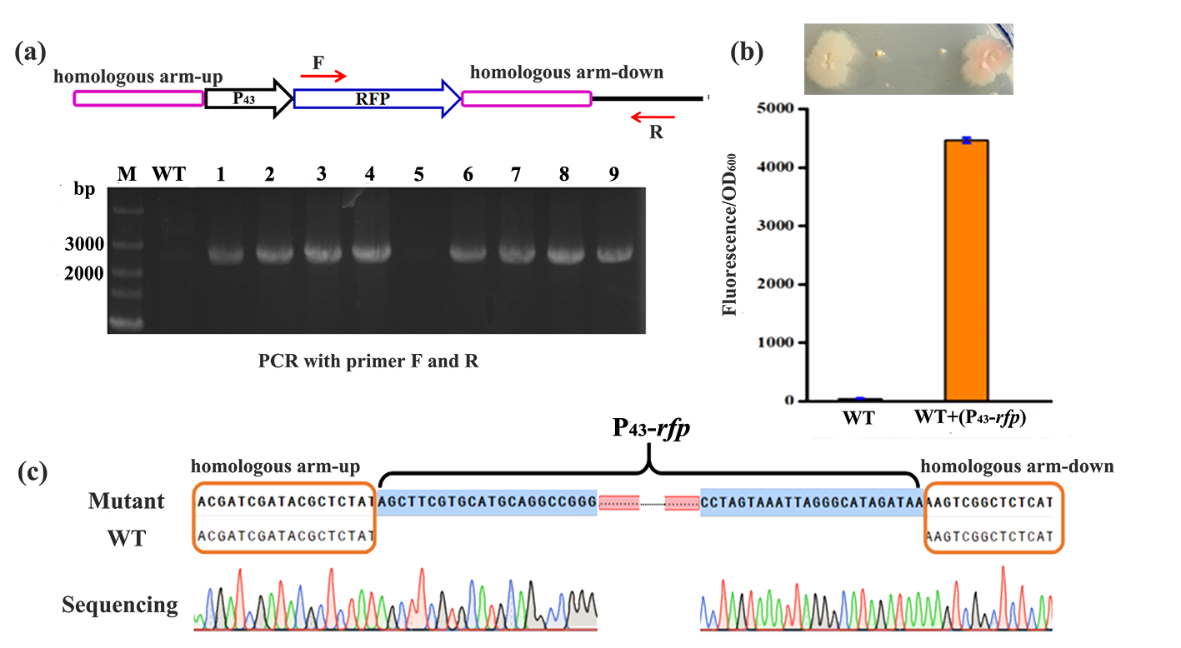


**Fig. S5.** **The verification of *rfp* insertion into genome of *Bacillus* sp*.* N16-5.** **(a)** Colony PCR of random selected 9 colonies; **(b)** The colony phenotype identification and RFP fluorescence value of *Bacillus* sp. N16**-**5 (wild-type control) and *Bacillus* sp*.* N16-5::P_43_-*rfp*; **(c)** Sanger sequencing of positive colonies.

**References**

A.Sullivan M, E.Yasbin R, and E.Young F. (1984) New shuttle vectors for *Bacillus subtilis* and *Escherichia coli* which allow rapid detection of inserted fragments. *Gene* **29**: 21-26.

Lu Z, Yang S, Yuan X, Shi Y, Ouyang L, Jiang S*, et al.* (2019) CRISPR-assisted multi-dimensional regulation for fine-tuning gene expression in *Bacillus subtilis*. *Nucleic Acids Res* **47**: e40.
